# The Memory Gene, *Murashka,* Is a Regulator of Notch Signalling and Controls the Size of the *Drosophila* Germline Stem Cell Niche

**DOI:** 10.3390/biom15081082

**Published:** 2025-07-26

**Authors:** Thifeen Deen, Hideyuki Shimizu, Marian B. Wilkin, Martin Baron

**Affiliations:** School of Biological Sciences, Faculty of Biology Medicine and Health, University of Manchester, Oxford Rd., Manchester M13 9PT, UK

**Keywords:** Notch, murashka, ubiquitin ligase, *Drosophila*, development, oogenesis, stem cells, niche

## Abstract

We identified Murashka, a RING finger protein, in an oogenesis screen as a regulator of *Drosophila* ovary germline stem cell niche development. Mutant alleles of *murashka* exhibited an enlarged niche phenotype reminiscent of increased Notch signalling and displayed genetic interactions with *Notch* alleles, and with *polychaetoid*, a regulator of Notch during niche development. These interactions uncovered both positive and negative impacts on Notch in different genetic backgrounds. In S2 cells, Murashka formed a complex with Notch and colocalised with Notch in the secretory pathway. Murashka expression in S2 cells down-regulated Notch signalling levels but could result in increased fold induction due to the proportionally greater decrease in basal ligand-independent activity. In vivo Murashka expression had different outcomes on different Notch target genes. We observed a decrease in the expression of *vestigial* along the anterior/posterior boundary of the wing imaginal disc, but not of *wingless* at the dorsal/ventral boundary. Instead, weak ectopic *wingless* was observed, which was synergistically increased by the coexpression of Deltex, a positive regulator of ligand-independent signalling. Our results identify a novel developmental role for *murashka*, a gene previously only associated with a function in long-term memory, and indicate a regulatory role for Murashka through a physical interaction with Notch that has context-dependent outcomes. Murashka adds to a growing number of ubiquitin ligase regulators which interact with Notch at different locations within its secretory and endocytic trafficking pathways.

## 1. Introduction

Notch is a 300 kDa transmembrane protein with a large extracellular domain, which is trafficked to the cell surface and modified in the endoplasmic reticulum (ER) and Golgi by Furin-mediated cleavage and addition of O-linked carbohydrate. These processes facilitate protein folding and transport to the cell surface, and determine specificity for different DSL domain (Delta/Serrate/Lag2) family ligands [1]. Ligand binding results in the removal of the bulk of the extracellular domain (ECD) through proteolytic cleavage at the S2 site by a metalloprotease [2]. This is coordinated with the transendocytosis of the detached ECD, in complex with the ligand, into the ligand-bearing cell [3]. The resulting membrane-tethered Notch intracellular domain (NICD) is subsequently cleaved at an intramembrane S3 site by the Presenilin-containing γ-secretase complex. Once released, the soluble NICD is trafficked to the nucleus, where in a complex with the transcription factor Suppressor of Hairless (Su(H)), it activates target gene transcription [4,5]. Endocytic trafficking plays a role in both activating and down-regulating the Notch receptor. It has been shown that the sorting of Notch between distinct endocytic compartments determines whether Notch is activated or down-regulated [6,7,8,9]. Numerous E3 ubiquitin ligases interact with Notch and regulate its signalling at different steps. The ubiquitination of the C-terminus of Notch by Sel10/FBXW7 (F-box and WD repeat domain containing protein-7) controls NICD levels through proteosome-dependent degradation [10,11]. Deltex (Dx) is a Ring finger ubiquitin ligase that binds NICD to promote the endocytosis of full-length receptor and regulate its trafficking to the limiting membrane of the late endosome/lysosome. The latter is associated with a Dx-induced, ligand-independent activation of Notch [7,8,12]. Suppressor of deltex (Su(dx)) is a HECT domain E3 ubiquitin ligase of the Nedd4 family, which also binds NICD and diverts endocytosed Notch into the internal lumen of the late endosome, associated with the down-regulation of Notch signalling [6,7,8]. The latter down-regulatory activity depends on the ubiquitination-dependent transfer of Notch to endosomal intraluminal vesicles, mediated by the Su(dx) HECT domain, which is temperature-dependent. In the absence of ubiquitination, Notch is activated from the endosomal membrane in a basal activation mechanism, even if Dx is not present [8]. This temperature-dependent switch acts to compensate for the effects of environmental temperature changes on ligand-dependent Notch signalling during development [8].

During *Drosophila* ovary development, Notch signalling is involved in the recruitment of cap cells to form the niche for the germline stem cells (GSCs) at the anterior tip of the germarium region of the ovary [13]. Notch is also required for maintenance of this GSC niche in the adult fly [14,15], and the loss of Notch signalling results in a reduction in the number of cap cells. In contrast, the overexpression of an activated form of Notch using C587 Gal4 results in the increased recruitment of cap cells into the niche and increased GSC numbers [13]. Similarly, the mutation of *polychaetoid* (*pyd*), the *Drosophila* homologue of the junctional protein ZO-1, results in an expansion of the cap cell niche through increased Notch signalling [16,17]. Cap cells express Decapentaplegic (DPP), which signals to the adjacent GSCs to promote their proliferation and self-renewal [18,19,20], and so an increased cap cell niche is expected to support additional GSCs, and a reduced niche to be occupied by fewer GSCs [13,15,16].

Here we identify several non-complementing mutations in *murashka* (*mura*), a gene encoding a RING (Really Interesting New Gene) finger domain protein, which result in an expanded number of cap cells in the GSC niche of the *Drosophila* ovary and show that *mura* is expressed in the cap cell niche during pupal ovary development. We demonstrate genetic interactions between *mura* and *Notch* alleles affecting GSC niche development and physical interactions between Mura and Notch in cell culture, where the proteins colocalise in the secretory pathway. Furthermore, we show that *mura* loss or gain of function affects Notch target gene expression in vivo and in vitro. Our results thus identify unexpected, Notch-associated, developmental roles for *mura*, a gene previously linked with long-term memory formation [21,22,23].

## 2. Materials and Methods

### 2.1. Drosophila Stocks and Culture

All experiments were performed at 25 °C on standard *Drosophila* culture medium unless stated otherwise. *mura*^EY03448^, *mura*^KG04118^, and Df(3R)BSC476 were obtained from the Bloomington Stock Center (University of Indiana, Bloomington, IN, USA). *mura^(GSV1)GS3176^* and *mura^(GSV6)GS10322^* were obtained from the Kyoto *Drosophila* Stock Centre (Kyoto Institute of Technology, Kyoto, Japan). The *mura*RNAi (GL00121; valium 22) stock was obtained from the TRIP stock centre (Harvard University, Boston, MA, USA). The expression of the RNAi and UAS constructs was performed using the GAL4 system [24]. Dpp-GAL4 [25] and ptc-GAL4 driver [26] were used to drive the expression of a UAS-mura cDNA construct in the wing imaginal disc. The germarium somatic cell driver C587-GAL4 [27] was used to express in the developing niche cells. The Gal4 enhancer-trap lines PGal4*mura^1^* and P(GawB)*mura^NP5945^* were obtained from Josh Dubnau (Department of Neurobiology and Behavior, Stony Brook University, Stony Brook, NY, USA) and the Kyoto Stock Center, respectively. For testing genetic interactions, we used UAS-Dx [12], *N*^55E11^ [28], *pyd*^147^ [16], and *N*^Ax-*E2*^ [29]. Vg^BE^-LacZ [30] was used as a reporter for Notch activity in wing imaginal discs. For transposon excision, the P(Δ2-3)99B/Tm6b stock was obtained from the Bloomington Stock Centre. The wild-type strain used for comparison is Oregon-R (OreR).

### 2.2. Molecular Biology

#### 2.2.1. Primers

MF1 (5′ GTCACGTCGAACCCAAGGTA); MR1 (5′ CATGTGTCTGTTGGCAGCTC); McDNAF1 (5′ CCAACTATCACCCGATGC); MXBAIV5TAG (5′ ATGCTCTAGACTGGCCGCTTGGCTCTGCTGTG GATTGGCGG).

#### 2.2.2. PCR of Genomic DNA

*Drosophila* genomic DNA was extracted from around 20 WT adult flies and crushed using a pestle and mortar in 500 μL homogenisation buffer (1 mL 1 M Tris (pH 8), 100 μL 5 M NaCl, 1 mL (0.5 M) EDTA (pH 8), 7.9 mL water). A total of 55 μL of 10% sodium dodecyl sulphate (SDS, Sigma-Aldrich, St. Louis, MO, USA) was added and mixed to enable permeabilisation. In total, 10 μL of RNAaseA (Sigma-Aldrich, St. Louis, MO, USA) was then added, and the samples were incubated for 30 min at 37 °C. A total of 10 μL of protineaseK (Sigma-Aldrich, St. Louis, MO, USA) was then added, and the sample was incubated for 2 h at 55 °C. The samples were then centrifuged (13,000× *g*) and the supernatant was transferred to a phase lock tube (Eppendorf UK, Stevenage, UK). DNA was extracted using a standard phenol–chloroform extraction, and DNA was precipitated by adding 1/10 of the sample volume of 3 M Sodium Acetate (pH 5.2, Sigma-Aldrich, St. Louis, MO, USA) and 2 sample volumes of 100% ethanol (Sigma-Aldrich, St. Louis, MO, USA). The sample was centrifuged at 13,000× *g* for 5 min, and the resulting pellet was washed twice with 70% ethanol. The pellet was then air dried and resuspended in sterile water (Sigma-Aldrich, St. Louis, MO, USA) and stored at −20 °C. PCR reactions were performed using Taq DNA polymerase (Qiagen, Manchester, UK) to amplify across the region of the P-element insertion using primers MF1 and MR1. The amplified DNA was separated using gel electrophoresis, and the appropriate band was extracted from the gel and sequenced using the Big Dye Terminator v3.1 Cycle sequencing kit (Applied Biosystems, Foster City, CA, USA).

#### 2.2.3. Construction of pUASTmura and pMTmura-V5 Vectors

cDNA for mura (LD30050, encoding isoform B) was obtained from the *Drosophila* Genomic Resources Center, (DGRC, Indiana University, IN, USA), restriction digested with BglII and XhoI, and the cDNA fragment was inserted into the BglII and XhoI-cut pUAST vector [24] to generate pUAST-mura. Transgenic fly lines were generated by BestGene Inc. (Chino Hills, CA, USA). The line used in this work was UAST-Mura^8M^, located on the third chromosome. To generate pMT-MuraV5, an XbaI site was first introduced using PCR (Primers McDNAF1 and MXBAIV5TAG) and the stop codon in the mura cDNA was replaced. The PCR fragment and pMT were digested using single-cutting enzymes NotI and XbaI. This produced a 3′ 0.75 kb fragment, which was cloned into pMT in frame with a C-terminal V5-tag. A 2.5 kb EcoRI Fragment from mura cDNA was then cloned into the 3′ mura fragment in pMT to generate pMT-mura-V5.

### 2.3. Cell Culture Experiments

#### 2.3.1. Luciferase Assays

*Drosophila* S2 cells (Thermo Fisher Scientific, Waltham, MA, USA) were cultured in Schneider’s *Drosophila* medium (Lonza, Slough, UK) supplemented with 10% heat-inactivated foetal bovine serum (Thermo Fisher Scientific) and 0.5% Penicillin–Streptomycin (Sigma-Aldrich) at 25 °C. Effectene (Qiagen) was used for transfection. For basal and Dx-induced signalling assay, the cells were transfected in a 12-well plate with pMT-Notch plasmid [8], NRE:Firefly (Sarah Bray, University of Cambridge, UK), and Actin:Renilla, either with or without pMT-Dx [8], and after 24 h cells were then transferred to a Nunc 96-well assay plate. Expression was induced by CuSO_4_ addition (1 mM final concentration). Signalling was assayed after a further 24 h using the Dual-Glo Luciferase assay system (Promega, Southampton, UK). A luminometer (Berthold, Bad Wildbad, Germany) was used to quantify Luciferase activity, and this was normalized with the Renilla value. For ligand-induced signalling, 2 days after transfection, CuSO_4_ was added directly to the 12-well dish before re-seeding cells into white 96-well plates on top of fixed (Dl expressing S2 cells (S2-Mt-Dl; DGRC) or control S2 cells. Fixation was 4% formaldehyde (Polyscience, Warrington, PA, USA) for 25 min, followed by washing 2X in PBS and 2X in Schneider’s medium/10% FBS). The Dual Glow assay was performed after 24 h of exposure to the ligand. For comparison of the impact of Mura on Notch, relative signalling data was normalised to the appropriate control condition, i.e., without Mura expression.

#### 2.3.2. Co-Immunopreciptiation

For co-immunoprecipitation, S2 cells were grown in 6 well dishes with 2 wells per sample (4 mL per sample) in Schneider’s medium at 25 °C and transfected with pMT-Gal4 and a combination of the following constructs pMT-Notch, pMT-Notch-GFP [9] and pMT-muraV5, Protein expression was induced using CuSO_4_ (1 mM final concentration) for 24 h. Cells were then homogenised in lysis buffer (50 mM Tris–HCl, pH 8.0, 150 mM NaCl, 1% Triton X-100, 1 mM CaCl_2_) and protease inhibitor cocktail (EDTA-free Complete; Roche, Basel, Switzerland), and cleared by centrifugation. The resulting lysates were incubated with GFP-trap (ChromoTek, Planegg, Germany) for 2 h at 4 °C. After several washes in lysis buffer, bound proteins were eluted, and western blotting was performed using mouse anti-V5 (Serotec, Kidlington, UK, 1/2000), mouse anti-Notch^intra^ (Developmental Studies Hybridoma bank (DSHB), University of Iowa, IA, USA, 1/10,000) and mouse anti-Pnut (DSHB, 1/10,000).

#### 2.3.3. Immunofluorescence Staining of S2 Cells

Coverslips were prepared by coating with 0.01% (*w*/*v*) poly-L-lysine (Sigma Aldrich, St. Louis, MO, USA) for 10 min at room temperature. They were then washed with water twice, followed by 100% ethanol. Coverslips were dried in a clean 6-well dish. The cells were transferred onto coverslips in the 6-well dish and left for 1 h to enable the cells to adhere. Media was removed and the cells were washed with 1XPBS. The cells were then fixed in 4% formaldehyde (Polyscience) for 30 min at room temperature. The cells were washed 1X with PBS and then permeabilised in 0.2% PBS-Triton X-100 (Sigma-Aldrich) for 15 min at room temperature. The cells were then blocked in 5% skim milk (Sigma-Aldrich) in PBS for 10 min before the addition of primary antibodies in blocking solution for 2 h and washed in PBS before the addition of secondary antibodies at room temperature in the dark for two hours The cells were washed in PBS and coverslips mounted on slides with Vectorshield-4,5-dianidino-2-phenylindole (DAPI) (Vector Laboratories, Newark, CA, USA).

For the antibody uptake assay, the cells were transferred to coverslips, as above, and washed once with Schneider’s medium at 4 °C. Anti-Notch-ECD antibody was added at a concentration of 1/200 in media. The cells were incubated for 15 min on ice. The cells were washed with culture media and then incubated for a further 1 h at 25 °C. The cells were then washed with 1xPBS and fixed, permeabilised and stained as above.

Primary antibodies rabbit anti-V5 (Bethyl laboratories, Montgomery, TX, USA; 1/200), mouse anti-Notch^ECD^ (DSHB, 1/200), rabbit anti-GM130 (1/100, Abcam, Cambridge, UK), rabbit anti-COPII (1/100, Thermo Fisher Scientific), mouse anti-Proline Disulphide Isomerase (PDI) (1D3, 1/200, Enzo Life Sciences, Farmingdale, NY, USA), secondary antibodies were from Jackson Immuno Research Ely, UK. For the visualisation of additional organelle compartments, the cells were transfected with pUAS-EYFP-Rab7, pUAS-EYFP-Rab5, pUAS-EYFP-Rab11 combined with pMT-Gal4 [8], or pMT-Fringe-GFP [31].

Images were acquired using a Zeiss M2 Microscope (Carl Zeiss, Oberkochen, Germany) and Hammatsu Orca-ER camera (Hamamatsu Photonics, Hamamatsu City, Japan), and images were deconvolved using Openlab (Improvision, Perkin Elmer, Warrington, UK) and 3 nearest neighbours, and processed using the Photoshop CS5 (Adobe, San Jose, CA, USA) software.

### 2.4. Drosophila Tissue Dissection and Staining

#### 2.4.1. Ovary and Gut Dissection and Immunostaining

Ovaries from appropriate fly genotypes were dissected as follows. Each fly was pierced through the thorax with pins onto Sylgard (Dow Coring, Midland, MI, USA) plates and dissected in Grace’s insect medium (Sigma Aldrich). The ovaries were removed from the abdomen using forceps, and ovarioles were separated from the sheath using minutiens insect pins (Fine Science Tools, Foster City, CA, USA). The ovarioles were fixed in 4% formaldehyde (Polyscience) for 13 min. After fixation, the ovarioles were rinsed with PBS-TritonX-100 (PBS-Tx) (0.1% *v*/*v*) to enable the permeabilisation of the tissue. The ovarioles were then blocked with normal donkey serum (Jackson ImmunoResearch, West Grove, PA, USA) made with PBS-Tx (0.5% *v*/*v*) for 1 h to reduce background antibody staining. The ovarioles were then incubated with primary antibodies overnight at 4 °C. After incubation, the ovarioles were washed five times for 10 min each time with PBS-Tx (0.1%) before incubation with the secondary antibodies at room temperature in the dark for two hours. The samples were then washed five more times in PBS-Tx (0.1%) for 10 min each time. One drop of Vectashield-DAPI (Vector Laboratories) was added. The sample was incubated at 4 °C overnight before slide preparation. The primary antibodies were Mouse alpha-Spectrin (DSHB 1/20), Mouse LaminC DSHB 1/10) and guinea pig Coracle (Richard Fehon, University Chicago, IL, USA 1/10,000). For each genotype, cap cells were identified and scored using the expression of Coracle, which delineates the cap cell boundaries, which distinguishes cap cells from adjacent escort and terminal filament cells. Germline stem cells (GSC) were determined using a combination of alpha-Spectrin staining and location adjacent to cap cells. For visualisation of actin, after secondary antibody staining, tissues were incubated with 0.5% Alexa 647-Phalloidin (Thermo Fisher, Waltham, MA, USA) for 1 h at room temperature. The preparations were washed for 2 × 15 min in 0.1%PBS-Tween20 before mounting in Vectashield-DAPI (Vector laboratories).

For adult intestine, whole intestinal tracts were dissected out in ice-cold PBS and transferred to Eppendorf tubes containing 500 μL of Schneider’s *Drosophila* medium (Lonza, Slough, UK). The cell medium was removed, and the tissues were fixed in 4% paraformaldehyde in PBS medium for 20 min on a roller at room temperature. Fixed samples were washed 5x in PBS-Tx 0.1% and incubated with the permeabilisation/blocking solution of PBS-Tx 0.3% and 4% Normal Donkey Serum (Sigma-Aldrich) for 2 h at room temperature on a roller. Afterwards, they were rinsed with PBS/0.1% Tween20 (Sigma Aldrich), then incubated with primary antibody for 2 h in 100 μL PBS-0.1% Tween20 at 4 °C on a roller. They were washed 3 times for 15 min each with PBS/0.1% Tween20 on a roller. A total of 100 μL of secondary antibody solution in PBS/0.1% Tween20 was added at room temperature for 2 h, and then the washing step was repeated. After the final wash solution was removed, the tissues were mounted. Primary antibodies were guinea pig anti-Delta (GP581, used 1 in 4000, gift from Marc Muskavitch, University of Indiana, Bloomington, IN, USA) and rat anti-DECadherin (DCAD2, used 1:1000, DHSB, University of Iowa, IA, USA).

#### 2.4.2. Wing Imaginal Disc In Situ Hybridisation

Digoxigenin-labelled antisense cRNA probes for *wg* and *mura* were used for the in situ hybridisations. Larvae were dissected in 0.1% PBS-Tween20 (by turning the anterior half inside out. Larvae were fixed in 4% formaldehyde (Polyscience) in PBS for 30 min at room temperature. They were then rinsed once with 0.1% PBS-Tween20 and transferred to ice-cold 100% methanol and stored at −20 °C overnight. The samples were rehydrated in 1XPBS-Tween20. The samples were then fixed in fresh 4% formaldehyde/PBS for 5 min. Wing discs were then washed in 500 μL of hybridisation wash buffer (HWB, 50% formamide, 5 × SSC, 0.1% Tween, 0.1 mg/mL tRNA, 50 mg/mL heparin adjusted to pH 4.5 with 1 M citric acid) for 10 min at 70 °C. The sample was then prehybridised in 1 mL of hybridisation buffer (HB, HWB with 0.1 mg/mL tRNA, 50 mg/mL heparin for 1 h at 70 °C). The HB was removed, leaving 300 μL. The samples were hybridised overnight at 70 °C with antisense RNA probes labelled with digoxigenin (DIG) (Boehringer Mannheim, Germany). DIG-labelled antisense RNA strands were made by in vitro transcription by T7 polymerase from 1 μg of linearised plasmid cDNA. The probes were denatured at 85 °C for 5 min, and then transferred immediately onto ice for 2 min, and then added to the prehybridised wing disc samples. A total of 1 μL of DIG-labelled RNA probe was added to 20 μL of HB, and this was added to the prehybridised sample. The samples were incubated overnight at 68 °C. After incubation, two washes were performed for 20 min and 3 washes were performed for 10 min with heated HWB at 68 °C. The samples were then incubated with 1/1000 of alkaline phosphatase conjugated anti-DIG antibody (Boehringer Mannheim) in PBS-Tween20 for 1.5 h at room temperature. The samples were washed 5 times for 10 min each time in 1XPBS-Tween20. The samples were then washed in NMTT (0.1 M NaCl, 50 mM MgCl_2_, 0.1 M Tris pH 9.5, 0.1% tween-20) twice for 5 min each time. The samples were then transferred to watch glasses, and a staining solution, consisting of 10 mL NMTT with 200 μL NBT/BCIP solution (Roche, Basel, Switzerland), was added, and the samples were left in the dark for the stain to develop for 10 min. The reaction was then stopped by adding 1 mL of a 20 mM EDTA (Sigma Aldrich) solution made in PBS-Tween20. The samples were then further rinsed in 1XPBS-Tween20 and mounted in 90% glycerol in PBS.

#### 2.4.3. Wing Imaginal Disc X-Gal Staining

Third instar larvae were dissected in 1XPBS and fixed in 0.5% Glutaraldehyde (Sigma Aldrich) in PBS for 10 min and then washed in PBS. The preps were then stained for 40 min at 37 °C using a staining solution containing: 1 mM MgCl_2_, 6 mM K_4_FeIICn_6_, 6 mM K_3_FeIICn_6_, and 0.2% X-gal. The samples were then rinsed twice in 1XPBS and mounted in 90% glycerol (Sigma-Aldrich) in PBS.

#### 2.4.4. Adult Wing Mounting

Adult fly wings were dissected with forceps and placed on a microscope slide, washed once with isopropanol, and then mounted under cover slips with Garry’s Magic Mountant, i.e., approx. equal parts Canada Balsam and Methyl Salicylate (Sigma-Aldrich).

## 3. Results

**Figure 1:**
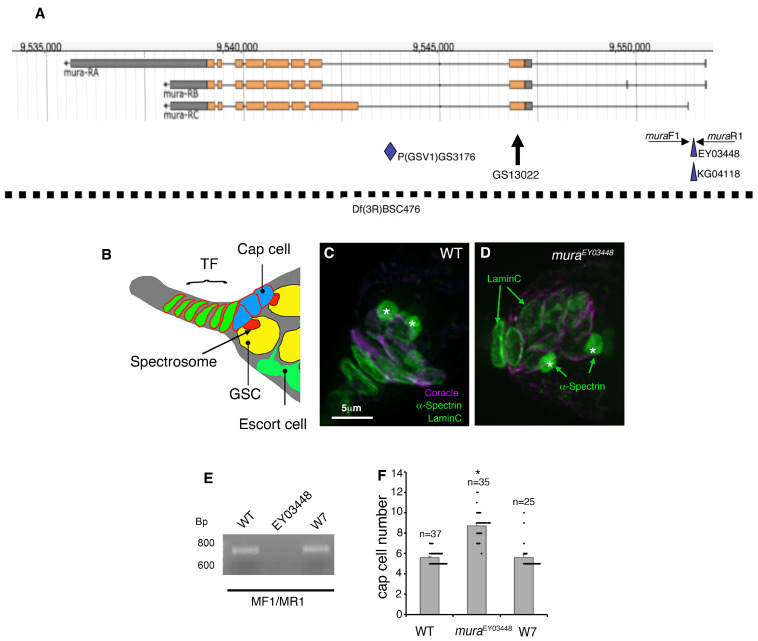
P-element insertion in *mura* results in an increase in the number of cap cells: (**A**) The *mura* gene locus (adapted from Flybase http://flybase.org/ accessed on 22 July 2025). Indicated are the positions of the P-element insertions used in this study. Orange rectangles indicate protein coding sequences. The dashed line indicates the deficiency used, Df(3R)BSC476. (**B**) Schematic representation of the structure of the niche. (**C**,**D**) Wild-type (WT) and *mura^EY03448^* germarium stained with anti-α-Spectrin, which marks the spherical spectrosomes of the GSCs (green—spherical structures indicated by the asterisks). Anti-LaminC (green—nuclear membranes) and anti-Coracle (purple) mark the cap cells. (**E**) PCR using primers muraF1 and R1 to confirm the precise excision of EY03448 P-element. A 720 bp band is observed when the P-element is absent. In flies where the P-element has been remobilised, this WT band is observed. (**F**) Scoring of niche size. Mean cap cell numbers are significantly different between WT and *mura^EY03448^*, but the phenotype is restored to WT following excision of the P-element. Error bars represent SEM, and * indicates *p* < 0.05 compared to WT by ANOVA with Tukey HSD test.

We performed a screen of homozygous viable P-element insertion lines to identify new genes involved in the regulation of oogenesis. We identified a P-element insertion mutation within the locus of the *mura* gene, EY03448 (Figure 1A), that displayed an expanded cap cell niche phenotype. The latter was visualised using anti-Coracle [32], which labels the cell boundaries of cap cells. LaminC staining was also performed, which labels the nuclear membranes of terminal filament and cap cells [33] (Figure 1B–D). The ovarioles were also stained with anti-αSpectrin to label the spherical spectrosomes, which identify the germline stem cells (GSCs) [34]. Surprisingly, *EY03448* females did not display an increase in GSC number compared to wild-type ovarioles (Figure 1C,D and Figure S1), indicating a disconnection between niche size and stem cell population size in this genotype. Other features of the germarium and early oogenesis appeared wild type.

To confirm that the insertion of the transposon element in the *mura* gene causes the observed cap cell niche phenotypes, the EY03448 P-element was remobilised by crossing in the Δ2-3 P-element transposase [35] to create a precise excision of the inserted element. Jump out events were selected for by the loss of the *white* eye colour marker, which is present in EY03448. Excision of the inserted P-element was then confirmed by PCR using primers that flanked the original insertion site (Figure 1A,E). Sequencing of the resulting PCR fragment revealed the jump out to be a precise excision. Precise removal of the inserted P-element restored the niche size to wild type (Figure 1F).

**Figure 2:**
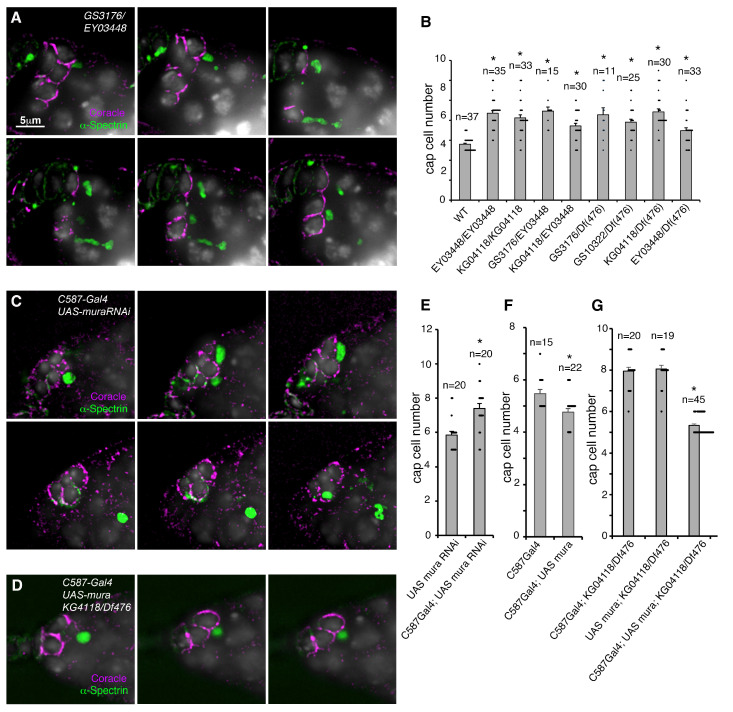
Loss and gain of function of *mura* are associated with the misregulation of niche size: (**A**,**B**) Complementation analysis of *mura* alleles. Germaria were stained with anti-Coracle (purple) to mark cap cells and anti-α-Spectrin to mark germ line stem cells and DAPI (greyscale) for nuclei. (**A**) depicts different z sections through the niche of a GSV3176/EY03447 germarium, showing an expanded niche comprising two clusters of cap cells on either side of the germarium. (**B**) Quantification of mean cap cell numbers across the different genotypes as indicated. * indicates *p* < 0.05 compared to WT, by ANOVA and Tukey HSD test. (**C**,**E**) Expression of the mura RNAi in the developing niche increases niche size. * indicates *p* < 0.05 compared to UAS-muraRNAi without Gal4, by *t*-test. (**F**) Expression of mura cDNA by C587 Gal4 reduced the size of the niche compared to the Gal4 driver alone. * indicates *p* < 0.05 compared to Gal4 driver alone by *t*-test. (**D**,**G**) Expression of mura cDNA rescues the *mura* mutant niche phenotype. * indicates *p* < 0.05 compared to C587Gal4; KG04118/Df(476) and UAS Mura; KG04118/Df(476), by ANOVA with Tukey HSD test. Error bars in (**B**,**E**,**F**) are SEM. WT data is also shown in Figure 1F.

To obtain further evidence that the disruption of the *mura* locus contributes to the niche size regulation, we identified additional transposable element insertions in the locus and performed complementation testing. In homozygous or heteroallelic combinations, all mutant alleles gave expanded niche phenotype, including when placed over deficiency BSC476 that removed the *mura* locus, confirming that the P-element inserts are loss-of-function alleles (Figure 2A,B). Most allelic combinations increased niche size without increasing GSC number, but we found the heteroallelic combination of GS13022/df(BSC476) also significantly increased GSC number (Supplementary Figure S1). Since GS13022 disrupts the ORF (Figure 1A) then this combination may be a stronger disruption of *mura* function.

The recovery of the wild-type phenotype following the remobilisation of the P-element confirms that the latter insertion in the *mura* locus causes the observed GSC niche phenotype, but is insufficient to show that it is the loss of function of *mura* itself that leads to the cap cell expansion. It is plausible that the mobile elements may also disrupt the function of nearby genes by insertion into a regulatory region. To confirm that loss of *mura* leads to the expanded niche phenotype, we used RNAi expression to knock down *mura* function using the C587 gal4 driver that was previously shown to express in the developing GSC niche [27,36]. The expression of *mura* RNAi phenocopied *mura* P-element allele phenotypes by producing an expanded niche phenotype when driven by C587 Gal4 (Figure 2C,E). There was also an increase in GSC numbers following RNAi knockdown (Supplementary Figure S1). We also expressed *mura* cDNA in the niche using C587 Gal4-driven expression [24], which slightly reduced the cap cell number compared to the Gal4 driver alone (Figure 2F) and also rescued the *mura* mutant phenotype (Figure 2D,G).

**Figure 3:**
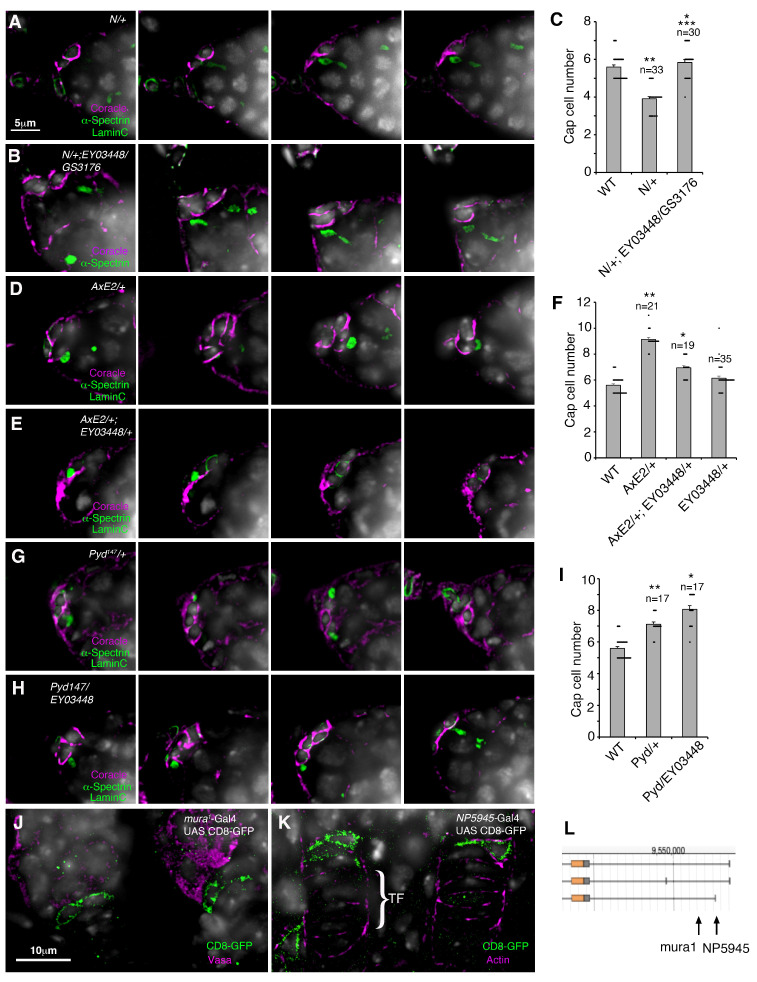
Genetic interactions between *mura* alleles and *Notch*: (**A**–**C**) Mutual suppression of *Notch/+* and *mura* mutant niche size phenotype. (**A**) Z sections of the reduced-size niche of *N/+* germaria. (**B**) *mura* mutant combination rescues the *N/+* phenotype. (**C**) Quantitation of *N/+* interaction with *mura* alleles. * indicates *p* < 0.05 compared with *N/+*, ** indicates *p* < 0.05 compared with WT, and *** indicates *p* < 0.05 compared with *EY03448/GSV3176;* compare this with Figure 2B. (**D**–**F**) Interaction between a *mura* allele and the *AxE2* allele of *Notch*. (**D**) *AxE2/+* germaria have an expanded niche size, which is partially reduced by one copy of the *mura* allele EY0448 (**E**). (**F**) Quantitation of genetic interaction between the *mura* and *AxE2* alleles. * indicates *p* < 0.05 compared with *AxE2/+*, ** indicates *p* < 0.05 compared with WT. (**G**–**I**) Genetic interaction between *mura* and *pyd*. (**G**) *pyd^147^/+* germaria have an expanded niche size, which is further increased by one copy of the *mura* allele EY0448 (**H**). (**I**) scoring of genetic interactions between the *mura* and *pyd* alleles. * indicates *p* < 0.05 compared with *Pyd^147^/+* and ** indicates *p* < 0.05 compared with WT. Error bars in (**C**,**F**,**I**) are SEM, statistics by ANOVA and Tukey HSD test. (**J**–**L**) Gal4 expression of CD8-GFP driven from the *mura* regulatory region in the developing niche during pupal ovary development. (**J**) *mura^1^*-driven Gal4 expression of CD8-GFP costained with anti-Vasa (purple), which labels germline cells, at 24 h after puparium formation. (**K**) NP5945-Gal4 expression of CD8-GFP costained with phalloidin to mark actin in the terminal filament (TF) of developing ovarioles, at puparium formation. (**L**) Location of Gal4 insertion sites in the *mura* regulatory region. Error bars in (**C**,**F**,**I**) are SEM. WT data in (**C**,**F**,**I**) is also shown in Figure 1F.

The specification and recruitment of cap cells during ovary development depends on Notch signalling. We therefore tested for genetic interactions between *mura* and *Notch* in the niche. As observed previously [17], loss of one copy of *Notch* reduced niche size and GSC number (Figure 3A,C and Figure S1). The loss of one copy of Notch was sufficient to reduce the *mura* expanded niche phenotype, showing that the latter phenotype depended on wild-type Notch activity (Figure 3C, compare with Figure 2B). The *mura* mutant combination also partially rescued the *N/+* mutant cap cell and GSC number phenotypes (Figure 3A–C and Figure S1). Intriguingly, we found that the *mura* mutation dominantly reduced an increased niche phenotype observed for the “gain of function” *Abruptex^E2^* (*AxE2*) mutant allele of Notch (Figure 3D–F). This suggests that in this *AxE2* genetic background, the overall contribution of WT Mura function is positive on Notch function and not negative. We also investigated the interaction between *mura* and the *Drosophila* ZO1 homologue, *polychaetoid (pyd).* The latter acts as a negative regulator of Notch, and *pyd* alleles cause an expanded niche phenotype through an increase in Notch signalling [16,17]. We found that a *mura* allele enhanced the dominant *pyd^147^* niche phenotype, consistent with a further increase in Notch activity (Figure 3G–I). In this genetic background, WT Mura appears to act negatively on Notch. The mutant phenotypes of *mura* are consistent with a normal role for *mura* to regulate Notch signalling in the cap cells, but contrasting outcomes indicate that this regulatory interaction is likely to be complex and context-dependent. To investigate *mura* expression in the developing ovary, we examined two different *mura* enhancer trap lines that express Gal4 to drive the expression of the membrane marker CD8-GFP. We found *mura* is expressed in cap cells during pupal ovary development (Figure 3J–L), consistent with a role in cap cell recruitment and differentiation. We also found the expression of *mura* in adult mid-gut intestinal stem cells and enteroblasts, suggesting a wider role in adult tissue homeostasis (Supplementary Figure S2).

**Figure 4:**
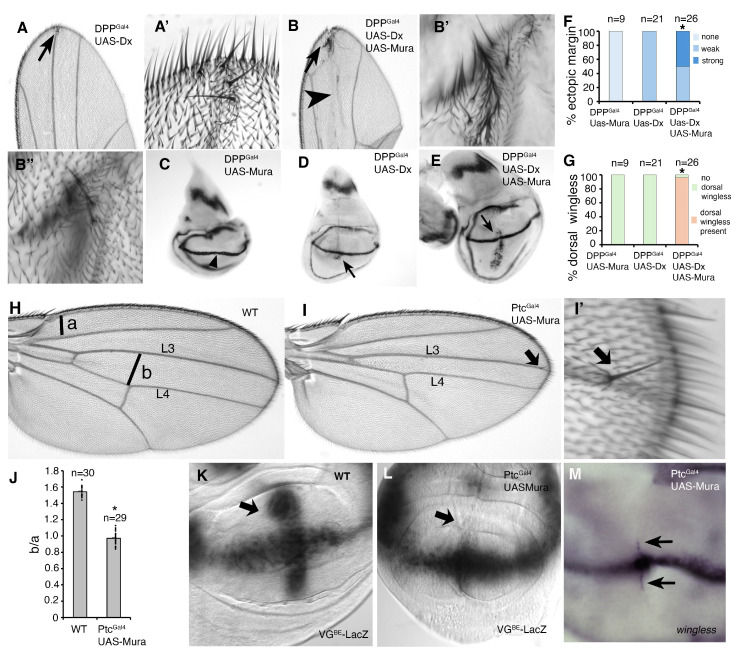
Ectopic Mura expression alters Notch signalling in wing development: (**A**) Weak ectopic wing margin phenotype observed when Dx is overexpressed with Dpp-Gal4, indicated by the arrow. (**A**′) Magnified image of the ventral ectopic margin in this region. Arrowhead indicates gaps in the L3 vein. (**B**) Strong ectopic wing margin phenotype following coexpression of Dx and Mura, indicated by the arrow. (**B**′) Magnified image of the ventral ectopic margin and (**B**″) dorsal ectopic margin. (**C**–**E**) In situ staining indicating *wingless* expression in late third larval instar wing imaginal discs, dorsal up, ventral below. (**C**) When *mura* is overexpressed with DPP-Gal4, this does not result in an increase in *wingless* expression. Endogenous wingless expression at the dorsal/ventral boundary is indicated with an arrowhead. (**D**) Weak ectopic *wingless* expression in the ventral compartment when Dx expression is driven by DPP-Gal4 is restricted to the ventral compartment, indicated by the arrow. (**E**) Coexpression of Mura with Dx results in increased *wingless* expression, which is observed in both the dorsal and ventral compartments of the wing disc, indicated by the arrow. (**F**,**G**) Quantitation of ectopic margin (**F**) and dorsal ectopic *wingless* expression (**G**) resulting from Dx and Mura coexpression. * in (**F**,**G**) indicates *p* < 0.05 by Chi-squared test. In (**F**), the weak category is similar to panel A and the strong category is represented by panel (**B**). (**H**) Wild-type female wing; the lines at a and b indicate distances quantified in (**J**). (**I**) Ptc-GAL4-driven Mura expression narrows the intervein region between the L3 and L4 longitudinal veins and results in a small number of ectopic bristles (arrow), shown in enlarged image (**I**′). (**J**) Quantification L3–L4 wing vein narrowing after Mura expression. The mean ratio of a/b was calculated for each genotype. * indicates *p* < 0.05 compared to WT by *t*-test. Error bars are SEM. (**K**,**L**) The vestigial boundary enhancer lacZ reporter in wild-type (**K**) and Ptc-Gal4 UASmura (**L**) flies. As indicated by the arrows, there is reduced vestigial expression of this reporter along the A/P axis of late 3rd instar wing imaginal discs compared to wild type. (**M**) *wingless* in situ. Ptc-Gal4-driven expression of Mura results in weak ectopic *wingless* expression in both dorsal and ventral compartments (arrows). Orientation of wing discs in (**C**–**E**,**K**–**M**) is dorsal up, ventral down.

To investigate whether the ectopic expression of Mura could regulate Notch, we used the DPP and Ptc-Gal4 drivers to express *mura* cDNA along the anterior–posterior (A-P) axis of the larval wing disc. We found that DPP-Gal4-driven Mura expression synergistically increased the activation of Notch by Dx, shown by increased ectopic margin in adult wings and increased *wingless* expression in late third larval instar wing discs compared to when Dx was expressed alone (Figure 4A–G). Notably, Dx alone induced *wingless* expression only in the ventral compartment of the wing disc, but when Dx and Mura were coexpressed, then *wingless* expression was also induced in the dorsal compartment (Figure 4D,E,G). *Mura* expression using the Ptc-Gal driver resulted in a narrowing of the inter-vein space between L3 and L4 (Figure 4H–J). This phenotype was associated with the loss of *vestigial* expression along the A-P axis of the wing disc (Figure 4K,L), as detected by the Notch-dependent vestigial boundary element enhancer linked to LacZ expression [30]. We found that Mura expression down-regulated VG^BE^-lacZ expression along the A-P axis but not where it crossed the D-V boundary (Figure 4L). In contrast, we did not observe any loss of expression of a second Notch target gene *wingless* at the D-V boundary of the wing, but instead observed weak ectopic *wingless* expression and occasional extra sensory bristles in the adult wings (Figure 4I′,M).

**Figure 5:**
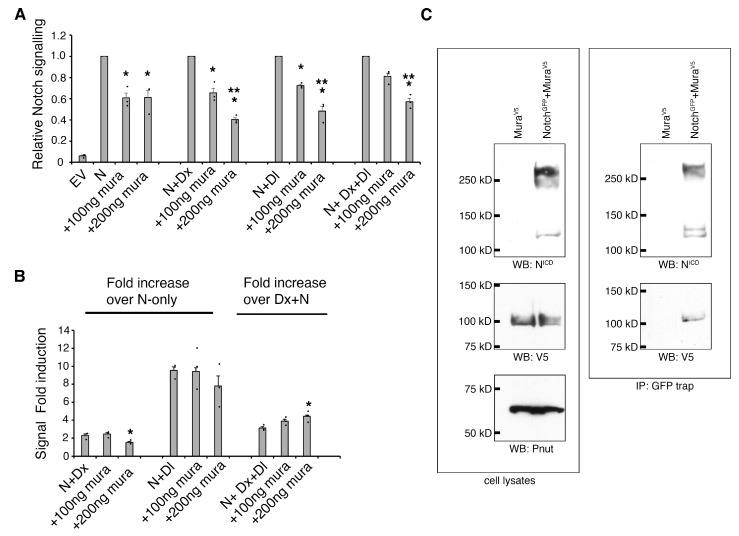
Mura regulates Notch in S2 cells: (**A**) Mura expression in S2 cells causes a dose-dependent reduction in both ligand-independent (basal and Dx-induced) and trans-ligand-dependent signalling levels. Mura also reduces activity when Dx and Notch-coexpressing cells are activated by the transexpressed ligand. EV indicates empty vector control. Signalling is measured by Notch response element-driven luciferase expression. For each experimental condition, Notch activity in the absence of Mura coexpression was normalised to 1. (**B**) Because of the reduction in ligand-independent signalling levels by Mura, the relative fold increase following ligand-induction is relatively stable or increases in a dose-dependent manner when both Dx-regulation and ligand-induced signalling are combined. Error bars in (**A**,**B**) indicate SEM, n = 3. * indicates *p* < 0.05 compared with the relevant no Mura control and ** indicates *p* < 0.05 compared to 100 ng mura, by ANOVA with Tukey HSD. (**C**) Notch and Mura form a complex detected by coimmunoprecipitation. Left panel—cell lysates; right panel—Notch-GFP precipitated using GFP trap. Western blots probed with either anti-Notch ICD antibody or anti-V5 to detect C-terminally V5-tagged Mura (Original images can be found in the Supplementary Materials (full gel images associated with Figure 5)).

To investigate the consequence of Mura expression on Notch activity further, we used an S2 cell culture luciferase-reporter assay for Notch signalling, which can be used to identify separate consequences on basal, Dx-induced, and trans-ligand-induced Notch activation [8]. The coexpression of Mura with Notch significantly reduced Notch signalling in a dose-dependent manner (Figure 5A). Notch signalling levels were reduced for both basal and Dx-induced trans-ligand independent signalling mechanisms. Mura also reduced Notch activation when stimulated by trans-ligand, with or without Dx-coexpression (Figure 5A). However, when considering the fold-increase by ligand stimulation, the fold induction compared to the absence of ligand was relatively stable when comparing Mura-expressing and nonexpressing cells (Figure 5B). In the context where both Dx and trans-ligand were present, there was a higher fold induction over the ligand-minus condition observed when Mura was coexpressed with Notch (Figure 5B) due to the proportionally greater reductions in ligand-independent activity.

**Figure 6:**
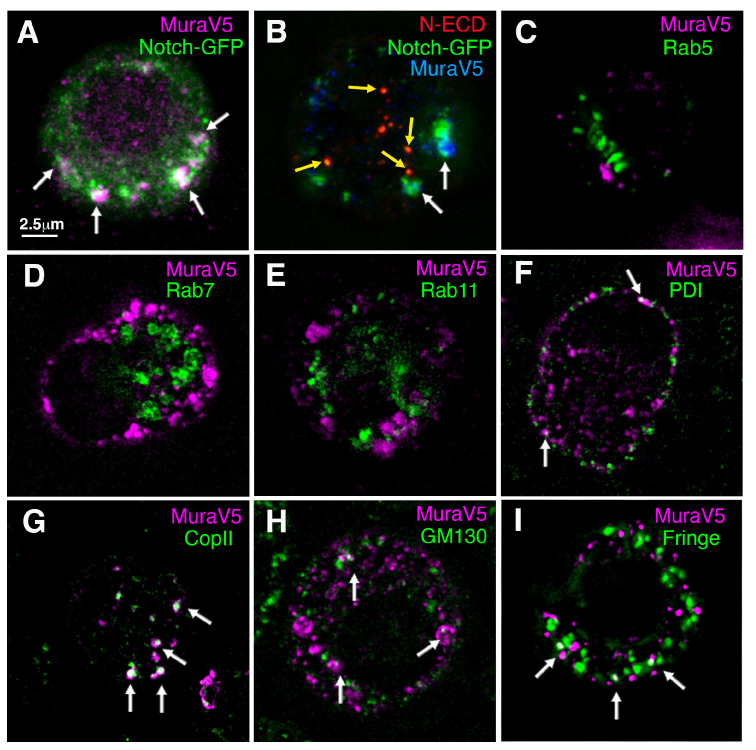
Localisation of Mura in S2 cells: (**A**) Notch-GFP and Mura-V5 show partial colocalisation when expressed in S2 cells. (**B**) After the endocytic uptake of Anti-Notch^ECD^ labelled surface Notch, there is colocalisation between anti-ECD and GFP-tagged ICD, indicating the endocytic uptake of full-length Notch. MuraV5 does not colocalise with ECD but does overlap with ICD-GFP lacking anti-ECD staining. (**C**–**E**) Mura-V5 does not colocalise with EYFP-tagged endosomal markers Rab5 (**C**), Rab7 (**D**), and Rab11 (**E**). (**F**–**I**) Puncta of Mura colocalisation with ER marker, PDI (**F**), intermediate compartment marker COPII (**G**), cis-Golgi marker GM130 (**H**), and trans-Golgi marker Fringe-GFP (**I**) are indicated with arrows. White arrows in (**A**,**B**) indicate examples of Mura-V5 colocalisation with Notch-GFP, yellow arrows in B indicate Notch anti-ECD localisation with Notch-GFP, white arrows in F-I indicate examples of Mura-V5 colocalisation with secretory pathway markers.

To determine whether Notch and Mura physically interact, we coexpressed a GFP-tagged Notch with V5-tagged Mura in S2 cells. Mura and Notch could be co-immunoprecipitated, indicating that Notch and Mura form a complex (Figure 5C). Consistent with this finding, Mura was found to co-localise with Notch in cytoplasmic vesicles (Figure 6A). To investigate whether Mura colocalises with Notch in the endocytic pathway, we expressed Notch with a GFP tag inserted into the ICD and performed a pulse-chase antibody uptake experiment on live cells with anti-Notch ECD to label Notch present at the cell surface during the labelling period. Dx was coexpressed with Notch to stimulate endocytosis. After a 60 min chase period, Notch-ECD staining colocalised in cytoplasmic puncta with GFP-tagged ICD (Figure 6B). However, Mura did not colocalise with this endocytosed Notch, but instead was found to be localised with GFP-tagged ICD in the absence of anti-ECD staining (Figure 6B). The results indicated that Mura is associated with Notch in the secretory pathway (not accessible to surface anti-Notch ECD immunostaining prior to its endocytosis), and not with endocytosed Notch. To determine where Mura is localised in the cell, we investigated its localisation compared with different compartment-specific markers. Consistent with the antibody uptake experiment, we did not see any colocalisation of Mura with the early, late, and recycling endosome markers EYFP-Rab5, EYFP-Rab7, and EYFP-Rab11, respectively (Figure 6C–E). We did, however, observe some partial colocalisation with secretory pathway markers (Figure 6F–I). PDI-marked endoplasmic reticulum showed infrequent spots of Mura localisation (Figure 6F); however, Mura was more clearly colocalised with COPII (Figure 6G), which marks the intermediate compartment between ER and Golgi [37]. Puncta of Mura colocalisation were also observed with the cis-Golgi marker GM130 and trans-Golgi localised Fringe (Figure 6H,I). The results indicate that Mura and Notch interact within the secretory pathway.

## 4. Discussion

### 4.1. Mura Regulates the Size of the GSC Niche

Pioneering work in the *Drosophila* ovary helped develop the niche concept of stem cell regulation, whereby stem cells reside in a defined location regulated by extrinsic factors derived from nearby cells [19,38]. An important signal that regulates the development of the ovary GSC niche is Notch, which is activated in the cap cells by its ligand Delta, expressed in the adjacent terminal filament cells. The latter signals to Notch during gonad development to recruit cap cells [13]. Increased Notch signalling in the developing niche results in ectopic cap cells, which support additional GSCs. Using temperature-sensitive mutants, it has been shown that Notch is also required in the adult to maintain both the niche and indirectly the number of GSCs [13,15]. We have previously shown that mutants in *polychaetoid*, the *Drosophila* homologue of the junctional protein ZO-1, result in increased cap cell and GSC numbers through increased Notch signalling [16,17]. During a screen of P-element insertion lines to identify mutations that resulted in early oogenesis phenotypes, we identified mutations in the gene locus of the *Drosophila* RING finger ubiquitin ligase-related protein, Murashka, which resulted in expanded numbers of cap cells. We confirmed the identity of *mura* as the gene affected through the remobilisation of the inserted P-element and failure to complement other *mura* alleles. Furthermore, *mura* RNAi expression in the niche resulted in similar phenotypes to *mura* mutants, while a UAS-*mura* cDNA expression construct expressed in the niche resulted in a reduced number of cap cells and rescued the *mura* mutant phenotype. These experiments also point to the cell-autonomous function of *mura* within the developing niche cells, and this was supported by the finding that enhancer trap GFP-reporter lines, inserted in the *mura* locus, expressed in nascent niche cells adjacent to the terminal filaments in pupal ovaries.

### 4.2. Functional Interaction Between Mura and the Notch Signalling Pathway

The *mura* expanded niche phenotype appeared similar to that expected from a gain of Notch signalling. Several lines of evidence suggested that *mura* and *Notch* functionally interact. Firstly, removing one copy of *Notch* rescues the *mura* phenotype and vice versa, showing that the expanded niche phenotype depends on normal Notch signalling. In addition, the *pyd* expanded cap cell phenotype, previously associated with increased Notch signalling [16,17], was dominantly enhanced by *mura* mutation. These results suggest that WT Mura may normally act to limit Notch activity, and this conclusion was supported by S2 cell culture experiments that showed that Mura expression down-regulates Notch signalling when measured by luciferase expression driven by a Notch response element. Both ligand-dependent and ligand-independent activation were down-regulated by Mura. However, other observations were indicative of a more complex regulatory outcome between Mura and Notch. Like *pyd* mutants, the *AxE2* mutation of Notch causes a dominant increased niche phenotype, but in the background of a heterozygous *mura* mutant, this phenotype was instead reduced. Similar opposing regulatory roles have been shown for other ubiquitin ligase regulators of Notch trafficking, Dx and Su(dx) [8,39]. More detailed consideration of the cell culture data provides hints regarding how Mura can have both positive and negative outcomes on Notch-dependent phenotypes. Because the basal level of ligand-independent signalling was proportionally the most affected by Mura expression, the fold-increase in activity was stimulated by the presence of Mura in conditions where Dx and Ligands were present. It is possible that in some in vivo regulatory contexts, the size of fold induction is more important than actual signal levels in determining the phenotypic outcome. This suggestion is, at present, speculative, however, and needs further work to explore its validity.

Intriguingly, the expanded niche of *mura* mutants did not result in proportionally more GSCs in most mutant allele combinations. Increased Notch signalling in GSCs themselves has been shown to promote GSC loss from the niche [40], and this may, therefore, account for reduced GSC to cap cell ratios in *mura* mutant germaria. This may explain why expressing mura RNAi only in the niche causes an increase in GSCs as well as an increase in niche size. One mutant allelic combination also increased GSC number, indicating that the precise nature of the gene disruption impacts the outcome, perhaps by having a differential effect on somatic and germ line cells, or by limiting the ability of the niche to promote expansion of GSC number through symmetric division. Further studies of *mura* functions in the germline stem cells may be informative in understanding the interplay between niche and stem cells to control niche occupancy.

In vivo overexpression experiments also provided contrary results as to the nature of the functional interaction between Mura and Notch, depending on which Notch target gene was being observed. When Mura was expressed in wing imaginal discs using the Ptc-Gal4 driver, there was suppression of *vestigial* expression along the A/P axis, which is a Notch-driven expression pattern, and this was associated with the narrowing of the adult wing between L3 and L4 veins. In contrast, we observed a small ectopic expression of another Notch target, *wingless,* which was associated with ectopic margin bristles. In addition, when Mura was coexpressed with Dx, we saw a large synergistic increase in ectopic *wingless* expression, indicating that in some regulatory contexts, Mura can act positively on Notch through Dx-induced signalling.

### 4.3. Notch and Mura Interact in the Secretory Pathway

Further support for a direct regulatory interaction between Notch and Mura came from the findings that the two proteins can participate in a complex in S2 cells, which was detected by co-immunoprecipitation experiments. We were also able to show colocalisation of the two proteins in intracellular compartments. Other ubiquitin ligases such as Su(dx) and Dx have been shown to interact directly with Notch in the endocytic pathway and alter Notch trafficking [6,7,8,9]. We could, however, find no evidence that Mura and Notch co-localise in the endocytic pathway, either from Notch antibody uptake experiments or by comparing Mura distribution with endocytic or recycling compartment markers. Instead, Mura showed partial colocalisation with ER and Golgi markers, suggesting a role within the secretory pathway.

Further work will be required to determine whether Notch is a ubiquitination target for the RING finger ubiquitin ligase domain of Mura. It is possible that such a modification may alter the trafficking of Notch with an impact on Notch signalling by different activation mechanisms. For example, previous work has shown that the diversion of membrane proteins from the secretory route to the late endosome/lysosome pathway is an alternative route for membrane proteins passing through the secretory pathway [41,42]. Since Notch is activated by its ligands at the cell surface, Mura might down-regulate ligand-induced Notch signalling by preventing Notch transport to the plasma membrane. Furthermore, by diverting Notch to the endosomes, it might be activated by the alternative Dx-promoted mechanism or down-regulated by lysosomal degradation. This could potentially explain the negative and positive effects of Mura on the expression of different Notch targets in different contexts. Alternatively, Mura may alter the ability of Notch to interact with various cofactors that are involved in forming the transcriptional activating complex with Suppressor of Hairless or may alter post-translational modifications affecting the ability of Notch to activate gene expression. It has previously been shown, for example, that different components of the transcription factor Mediator complex are required for either the repression or activation of different Notch-regulated targets, highlighting the importance of cofactor composition [43]. Post-translational modifications of the intracellular domain by methylation, acetylation, and ubiquitination have also been linked to the stability and capacity of the Notch intracellular domain for downstream activation of gene transcription [5,10,44,45]. Other modifications occurring in the secretory pathway include the addition of carbohydrate moieties to the Notch ECD in the ER and their further modification in the Golgi. These additions regulate Notch trafficking and also the capacity for signal induction through modulating ligand interactions. The Notch extracellular domain is modified in the endoplasmic reticulum (ER) by the addition of O-linked glucose through the activity of the O-glucosyltransferase, Rumi [46]. The latter is required in *Drosophila* to maintain sufficient Notch signalling at high fly culture temperatures. Notch is also modified in the ER by the addition of O-linked fucose by the O-fucosyltransferase, O-fut1, which acts as a chaperone protein, regulating Notch trafficking [47,48]. Fucosylation of Notch is also essential for Notch signalling [49]. The O-linked fucose moiety additionally acts as a substrate for further sugar chain extension by Fringe, which takes place in the Golgi body. Fringe modification of Notch prevents Notch activation by one of its ligands, Serrate, but facilitates Notch activation by Delta [50]. Further work will be needed to distinguish whether Mura affects any of these regulatory processes to impact Notch activity.

## 5. Conclusions

Mura has previously been identified as a mutation affecting long-term memory [21,22,23]. It has also been reported to be involved, along with a number of other RING finger ubiquitin ligase proteins, in regulating the immune deficiency pathway and anti-microbial response [51]. *Mura* mutants are homozygous viable; however, our studies now show that *mura* is also involved in developmental cell fate decisions. We did not observe other visible phenotypes in the morphology of the adult *mura* mutant flies, suggesting that Mura does not have a widespread developmental role. However, the finding that Mura is also expressed in intestinal stem cells and enteroblasts suggests other roles in adult homeostasis may be uncovered. It will be important, therefore, to explore Mura functions in other stem cell regulatory contexts. It is also interesting to note that, like Mura, Notch has also been shown to be involved in long-term memory formation [52,53], and the possible relationship between Mura and Notch signalling in this process should be explored. The human genome contains two genes that are related to *mura,* RNF38 and RNF44, which have been associated with both pro- and anti-tumour cell activity and drug resistance [54,55,56,57]. It will be interesting, therefore, to investigate if either of these Mura-related proteins also interacts with human Notch.

## Data Availability

The data and resources generated during this project are available upon reasonable written request.

## Supplementary Materials

The following supporting information can be downloaded at https://www.mdpi.com/article/10.3390/biom15081082/s1, Figure S1. Consequences of the *mura* mutation on GSC population size in the niche. Figure S2. Mura expression in *Drosophila* mid-gut
