# Supplementary material for: The Memory Gene, Murashka, Is a Regulator of Notch Signalling and Controls the Size of the Drosophila Germline Stem Cell Niche
Source: Biomolecules. 2025 Jul 26;15(8):1082. doi: 10.3390/biom15081082 (PMC12383540; doi:10.3390/biom15081082)
Supplement: Supplementary file 1 [file biomolecules-15-01082-s001.zip › biomolecules-3675791-supplementary.pdf]

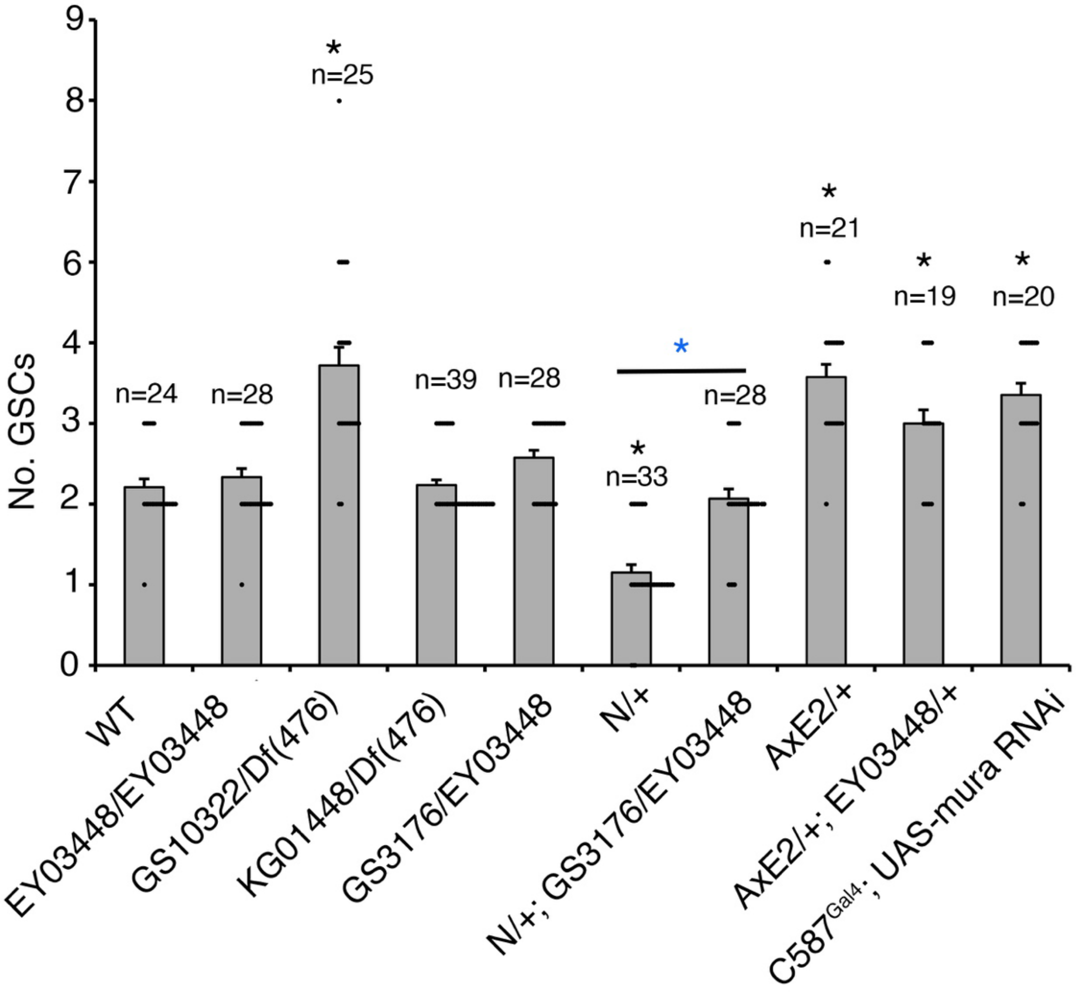

Supplementary Figure S1. Consequences of *mura* mutation on GSC population size in the niche. Combinations of *mura* alleles in WT and *Notch* mutant allele backgrounds. mutant combinations that had significantly different GSC number compared to WT ($p<0.05$) are indicated with black asterisk. Blue asterisks indicate $p<0.05$ between genotypes indicated by horizontal bars. Significance by ANOVA with Tukey HSD test. Error bars are SEM, sample sizes for each genotype are indicated on figure.

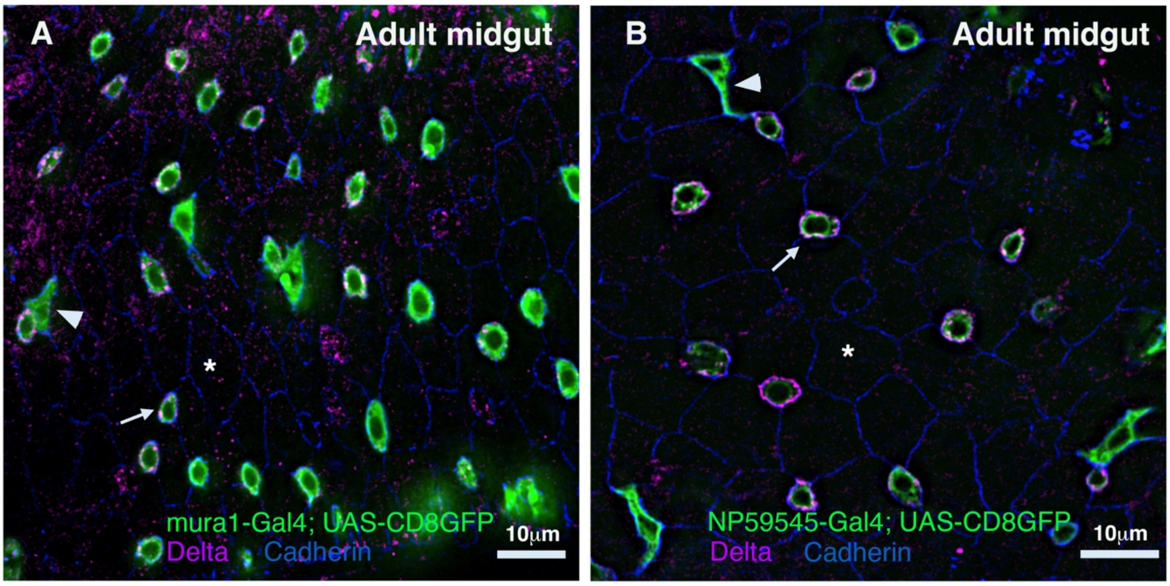

Supplementary Figure S2. Mura expression in *Drosophila* mid gut. Drosophila mid- guts were immunostained with anti-Delta (purple) to label intestinal stem cells (ISCs) and anti-Cadherin (blue) to mark all cell boundaries. Gal4 driven expression of CD8-GFP (green) marks mura expression using Gal4 enhancer trap constructs mura1 (A) and NP59545 (B). Arrows mark examples of mura expression in Dl-marked ISCs, arrow heads mark mura expression in enteroblast cells which are daughter cell products of asymmetric ISC division and * mark examples of large enterocyte cells which are the outcome of enteroblast maturation. Notch signalling promotes both enteroblast formation and maturation to enterocytes.

Full gel images associated with Figure 5

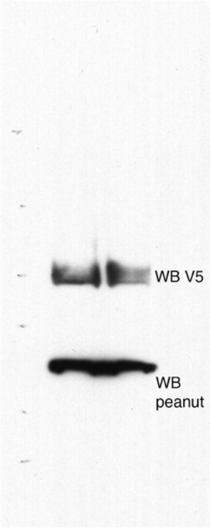

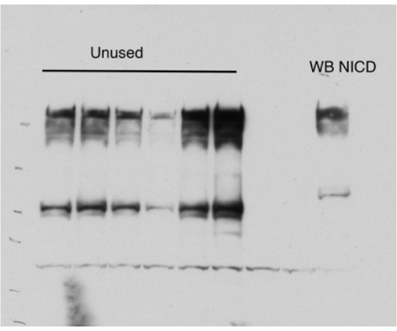

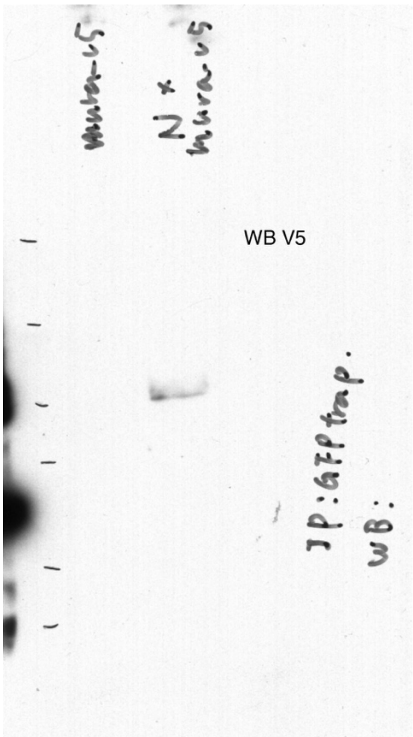

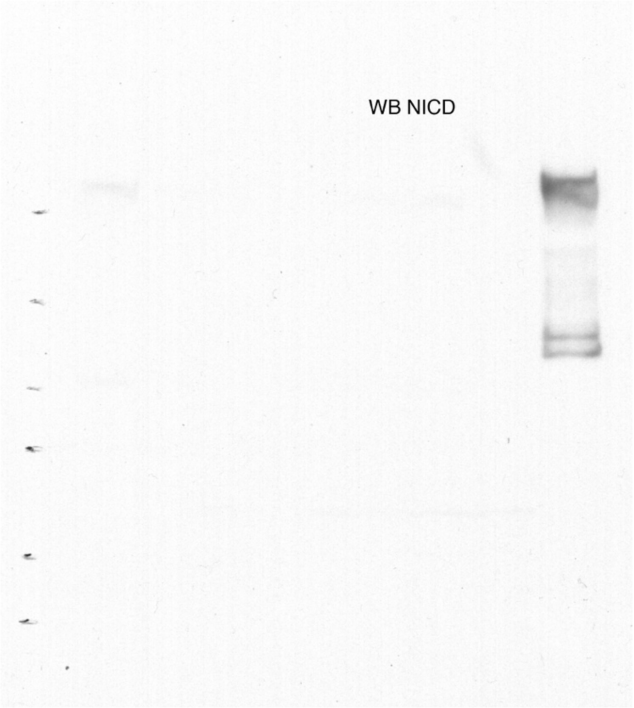
WB NICD
